# Antioxidant and anticancer activities of chamomile (*Matricaria recutita* L.)

**DOI:** 10.1186/s13104-018-3960-y

**Published:** 2019-01-03

**Authors:** Bayan Al-Dabbagh, Ismail A. Elhaty, Mohamed Elhaw, Chandraprabha Murali, Ameera Al Mansoori, Basma Awad, Amr Amin

**Affiliations:** 1 Department of Chemistry, College of Science, UAE University, PO Box 15551, Al Ain, UAE; 2 Department of Biology, College of Science, UAE University, PO Box 15551, Al Ain, UAE

**Keywords:** Antioxidants, Anticancer, Medicinal plants, Traditional medicine

## Abstract

**Objectives:**

The present study aimed at determining the antioxidant activity, total phenols and flavonoids and to evaluate the antiproliferative activity of ethanolic extract of *Matricaria recutita* L. (chamomile). The antioxidant activities were measured using the 2, 2-diphenyl-1-picrylhydrazyl (DPPH) assay. The total phenolic content was measured by the Folin–Ciocalteu assay. The flavonoid content was determined using the aluminum chloride method. The MTT assay was used to estimate the antiproliferative activities against human hepatoma (HepG2) cancer cell line. We assessed the mode of action of the extract as a cancer preventive agent and reported its ability to regulate tumor angiogenesis by down regulating in a dose dependent manner the expression of some proteins involved in the process.

**Results:**

The percentage inhibition of DPPH scavenging activity was dose-dependent ranging between (94.8% ± 0.03) at 1.50 mg/mL and (84.2% ± 0.86) at 0.15 mg/mL. It showed high polyphenols (21.4 ± 0.327 mg GAE/g) and high flavonoids content (157.9 ± 2.22 mg QE/g). Effect of extract was investigated against HepG2 cells. A dose-dependent reduction in cell viability was recorded in cells treated with the extract. The IC_50_ was ~ 300 µg/mL. It significantly inhibited the level of important prerequisite angiogenesis markers both in HepG2 cells and ex vivo.

**Electronic supplementary material:**

The online version of this article (10.1186/s13104-018-3960-y) contains supplementary material, which is available to authorized users.

## Introduction

The escalating knowledge of reactive oxygen species (ROS) has revolutionized medicine [1]. ROS contributes to cardiovascular diseases and cancer [2, 3] and could cause immune system depletion [3, 4].

A typical antioxidant donates an electron to a free radical thus neutralizing it [5] and often bind to metals [6]. Synthetic antioxidants cause negative health effects [7]. Reports have recommended to replace synthetic antioxidants with natural ones [6, 8] to control formation of free radicals [9]. The most common natural antioxidants are antioxidative enzymes [10] whereas others are well represented in different spices and herbs [11].

Chamomile is known for its as anti-inflammatory [12, 13], anti-diarrhea [14], antioxidant [14, 15], anti-cancer [16], neuro-protective [17], anti-allergic [18] and anti-microbial [19] effects. It also improves cardiac health [20].

Medicinal plants are essential to medicine [21, 22] where different preclinical studies using skin and ovarian cancer models have shown promising growth inhibitory effects [16, 23]. Similarly, chamomile was reported to induce apoptosis in cancer cells [24]. The main component of the essential oil extracted from chamomile is terpenoids α-bisabolol [25].

## Main text

### Methods

#### Plant sample and extract preparation

Dried chamomile flowers were purchased from the local market. Ten grams of the flowers were extracted with 70% ethanol and macerated for 48 h at 4 °C. The product was then filtered and concentrated under reduced pressure at 40 °C. A 30 mg/mL solution was prepared in 50% ethanol.

#### Total polyphenol content (TPC)

The Folin–Ciocalteu reagent was used [26]. 50% ethanol was used in the preparation of a 10% solution. Hundred µL of this solution was mixed with 200 µL of the Folin–Ciocalteu reagent and 2 mL of de-ionized water. 20% aqueous sodium carbonate (w/w, 1 mL) was added. After incubation for 1 h at room temperature, the total polyphenols were determined. Absorbance was measured at 765 nm. The total phenolic compounds were expressed in mg gallic acid equivalents (GAE) per g dry weight of plant material. Experiments were conducted in triplicates.

#### Free-radical scavenging activity

The 2, 2-diphenyl-1-picrylhydrazil (DPPH^·^) was used to measure the free radical scavenging activity [27]. 3.8 mL of 60 µg/mL methanolic solution of DPPH was mixed with 200 µL extract. This was allowed to stand for 30 min at room temperature in the dark. The absorbance was measured at 517 nm followed by the determination of the EC_50_ value (µg/mL). The total antioxidant activity was expressed as ascorbic acid equivalent/g dry extract. Experiments were conducted in triplicates.

#### Determination of total flavonoids

The aluminum chloride colorimetric method was used as described in [28]. 500 µL of the extract (600 µg/mL) was mixed with 0.1 mL of 10% (w/v) aluminum chloride, 0.1 mL of 1 M potassium acetate, 1.5 mL of methanol and 2.8 mL of distilled water. The mixture was incubated at room temperature for 30 min. The absorbance was measured at 415 nm. The mean of three readings was calculated and was expressed as mg of quercetin equivalents (QE)/g of the dry extract.

#### Cell culture

Human hepatoma cells (HepG2) were allowed to grow in RPMI 1640 medium (Sigma, USA) supplemented with 1 percent antibiotic cocktail and ten percent fetal bovine serum (FBS) [29]. Liver cells were keep warm at 37 °C in five percent CO_2_ humidified incubator then passaged every 2–3 days using 0.25% trypsin–EDTA solution.

#### Cytotoxicity assay

HepG2 cells were seeded at 5000 cells/well in 96-well plates (Nunc) where the tested extract was added in and its effect was assessed after 24 h. The 3-[4,5-dimethylthiazol-2-yl]-2,5-diphenyltetrazolium bromide (MTT) assay was applied to assess cell viability. Briefly, cells treated with the given extract were treated with 5 mg/mL tetrazolium MTT (Sigma, USA). The absorbance was then measured at a wavelength of 570 nm and the percentage of the viability of the treated cells was calculated using the following equation.

$$ Percent\;of\;viable\;cells = \frac{Abs\;of\;treated\;cells}{Abs\;of\;control\;cells} \times 100 $$

#### Assessment of morphological changes

HepG2 cells were seeded at 0.25 × 10^6^ cells/well in 6-well plates (Nunc) and were allowed to attach overnight. They were then treated without or with increasing concentrations of chamomile for 24 h and cell morphology was monitored and analyzed.

#### Experimental animals

The present study was approved by the institutional (UAE University) Animal Ethics Committee (approval Reference number: A 8-15) as detailed in [30].

#### Rat aorta ring assay

This was carried out on rat aortic explants as described [31]. Twelve male Wistar rats were used and approximately 12–14 rings were cut out from each aorta. The detailed methodology is described in [30].

#### Immunofluorescence

HepG2 cells were grown and processed for immunofluorescence as previously reported in [32].

#### Immunoblotting

HepG2 cells were seeded at a density of 1 × 10^6^ cells/100 mm plate and were then allowed to attach. Attached cells were treated with the given extract at the following doses (200, 400, 600, 800, 1000 µM) and for 24 h. Whole cell lysates were analyzed using 7.5% and 10% SDS polyacrylamide gel electrophoresis. Proteins were transferred onto PVDF membranes before incubation with primary antibodies (AKT, p-AKT, VEGF (Cell signalling technologies) Phospho-p44/42, Phospho-p38, MMP-9 (Abcam) and GAPDH (Abcam) as a loading control. All protein bands were detected using WesternSure Chemiluminescent Substrate (LI-COR) and C-DiGit blot scanner (LI-COR).

### Results and discussion

#### Plant extraction

The extract was prepared by evaporation of ethanol/water and the w/w percent yield was estimated to be 13.51% based on the used solvent [33].

#### Total polyphenol content (TPC)

Polyphenols are aromatic secondary plant metabolites [34] that may contribute to antioxidant activity [27, 35]. The Folin–Ciocalteu reagent reacts with polyphenols [36].

The calibration curve showed linearity for gallic acid in the range of 0.5–26 µg/mL, with a correlation coefficient (R^2^) of 0.988. Our extract contained high polyphenols (21.4 ± 0.327 mg GAE/g). When compared to other chamomile extracts, Italian chamomile gave (2689.2 ± 15 mg GAE/100 g) which is slightly higher than ours [37] while Egyptian chamomile showed (3.7 ± 2.0 mg GAE/g) polyphenols, much less than ours [33]. Results are the average of triplicates ± SD.

#### DPPH radical scavenging activity

Antioxidant activity was assessed based on the ability to reduce the stable DPPH radical [27]. The DPPH radical (DPPH·) can accept an electron or hydrogen radical and form a stable diamagnetic molecule [38]. The color change of DPPH has been used to measure the radical scavenging activity [39].

The ascorbic acid calibration curve showed linearity in the range of 5–20 µg/mL, with a correlation coefficient (R^2^) of 0.989. The DPPH scavenging activity of our extract ranged between (94.8% ± 0.03) at 1.50 mg/mL to (84.2% ± 0.86) at 0.15 mg/mL (Additional file 1).

The EC_50_ value was 26.7 µg/mL. We had a significant negative correlation (R^2^ = 0.856, P-value < 0.05) between EC_50_ ((DPPH·) scavenging) and total phenolic content. This suggests that their presence contributed significantly to antioxidant activity. These findings are consistent with previous investigations [27, 35, 40]. Iranian chamomile showed very high EC_50_ (5.52 ± 0.15 mg/mL) compared to our chamomile indicating that it has lower antioxidant power than the extract presented here [41].

#### Total flavonoid contents

Flavonoids have anticancer activity [42] and contribute to antioxidant plant properties [40]. We used the aluminum chloride assay [43]. The calibration curve showed linearity in the range of 1–25 µg/mL, with a correlation coefficient (R^2^) of 0.999. The total flavonoids content was (157.9 ± 2.22) mg QE/g dry extract.

#### Antiproliferative effect of *M. recutita*${\text{L.}}$ extract against cancer cells

HCC is among the leading causes of cancer death worldwide [44]. Natural products-based biomolecules possess bioactive secondary metabolites that are the foundation of broad-spectrum integrative approach for cancer prevention and treatment [45, 46].

**Fig. 1:**
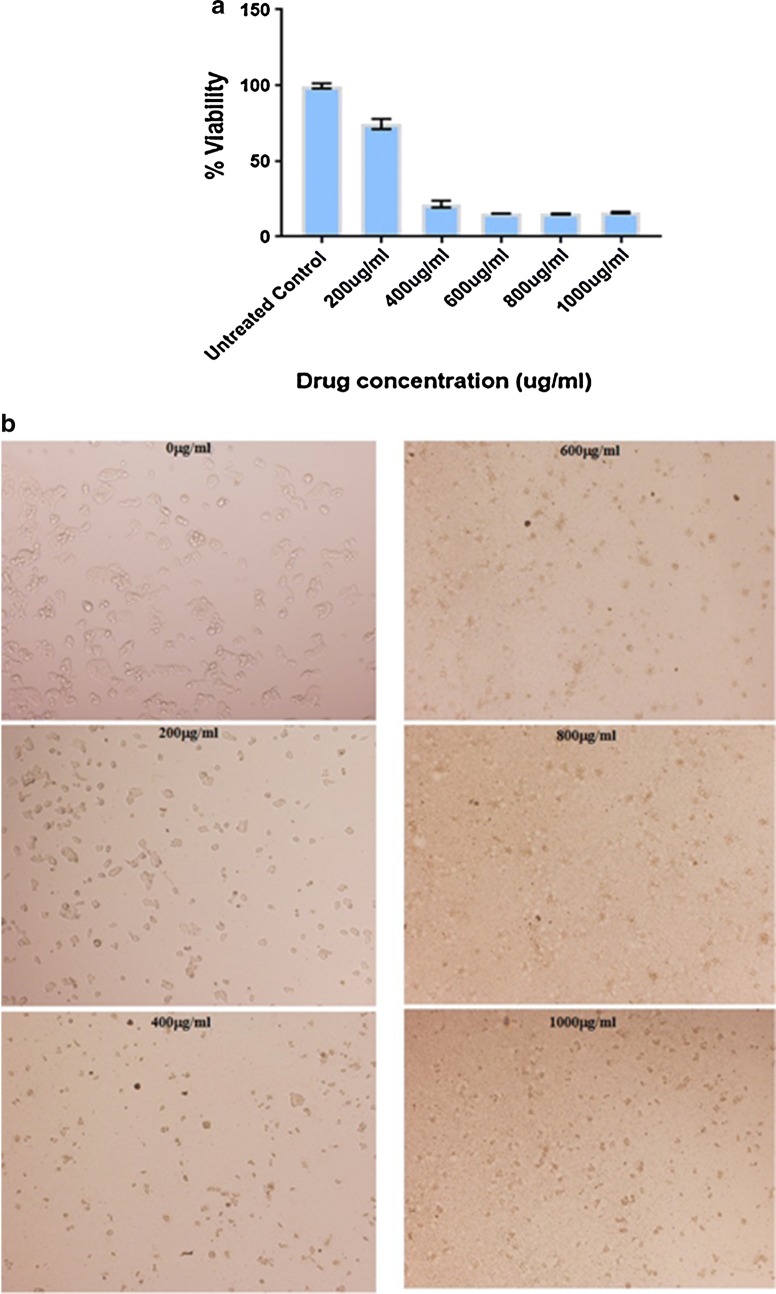
Assessment of the cytotoxic effects of *Matricaria recutita* L. extract on hepatocellular carcinoma in vitro. **a** MTT assay results of HepG2 cells viability after treatment with increasing concentrations of *M. recutita* L. for 24 h. **b** Assessment of morphological changes of HepG2 cells after treatment with increasing concentrations of *M. recutita* L. for 24 h. Cells were fixed and stained with crystal violet (scale bar = 200 μm)

Effects of the given chamomile’s extract were investigated in vitro against HepG2 cells. A dose-dependent reduction in cell viability was evident (Fig. 1). The IC_50_ value was 300 µg/mL and the extract significantly enhanced the mortality of cancer cells at concentration as low as (200 µg/mL). It is worth stating here that the present extract showed no hepatotoxicity in normal porcine liver primary cells as previously reported in [47]. Chamomile-based sesquiterpenic compounds have been reported to be involved in a plethora of biological activities [48].

Chamomile has many health promoting effects including anti-allergic and anticancer activities [49]. The composition and effects of chamomile have been studied; yet, the exact mechanism/s of its bioactivity awaits further investigations. Unlike the potent anticancer effect of the ethanolic extract of chamomile shown here, *M. recutita* L. infusion and plant methanol extract was selective for HCT-15 and HeLa and showed no activity against MCF-7, NCI-H460 and HepG2 [47].

#### Antiangiogenic activity of *M. recutita *${\text{L.}}$ extract in HepG2 cells

**Fig. 2:**
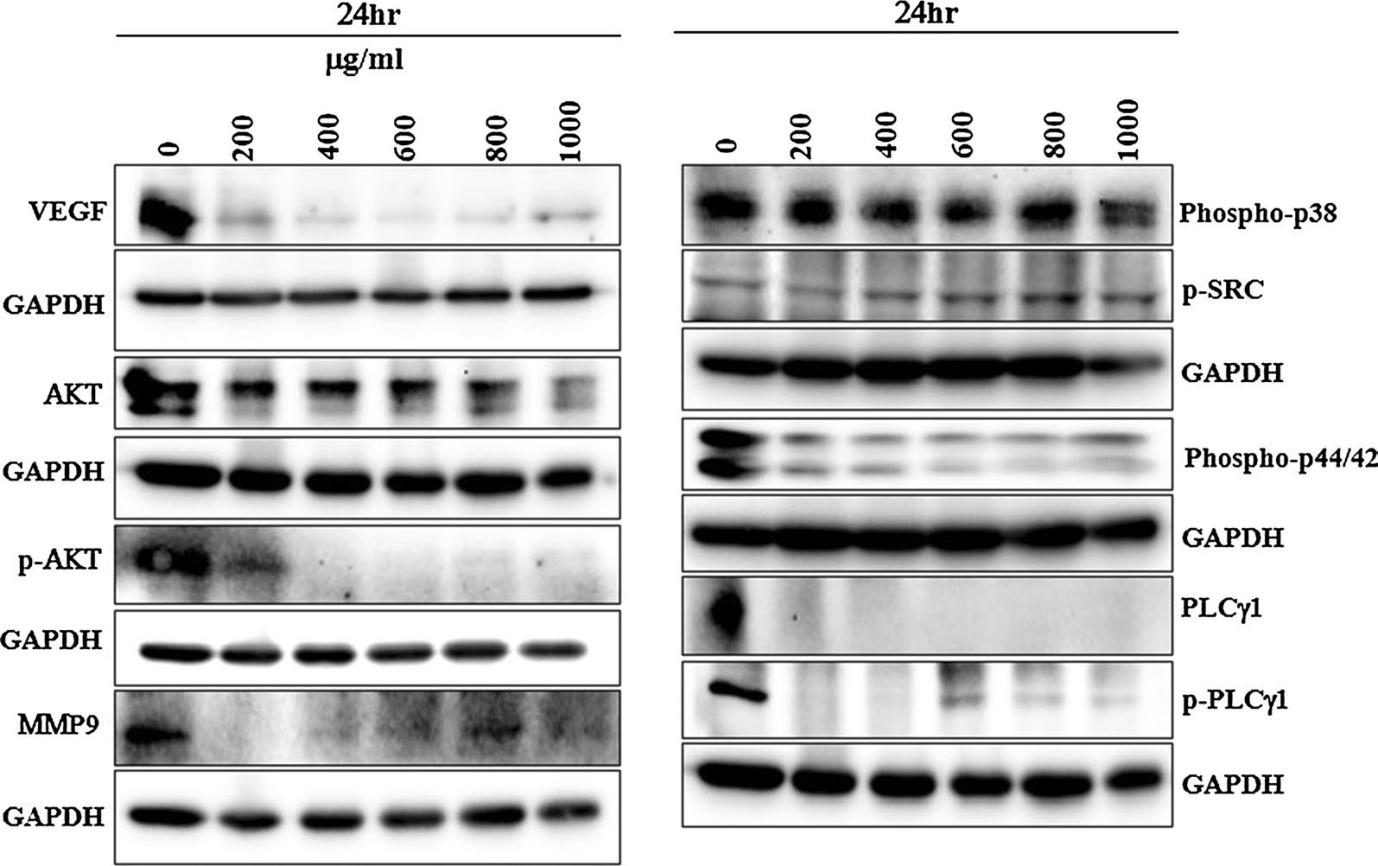
Inhibitory effect of *Matricaria recutita* L. extract on angiogenesis related markers. Western blot analysis of important and prerequisites markers in angiogenesis in HepG2 cells post treatment with increasing doses of chamomile for 24 h

**Fig. 3:**
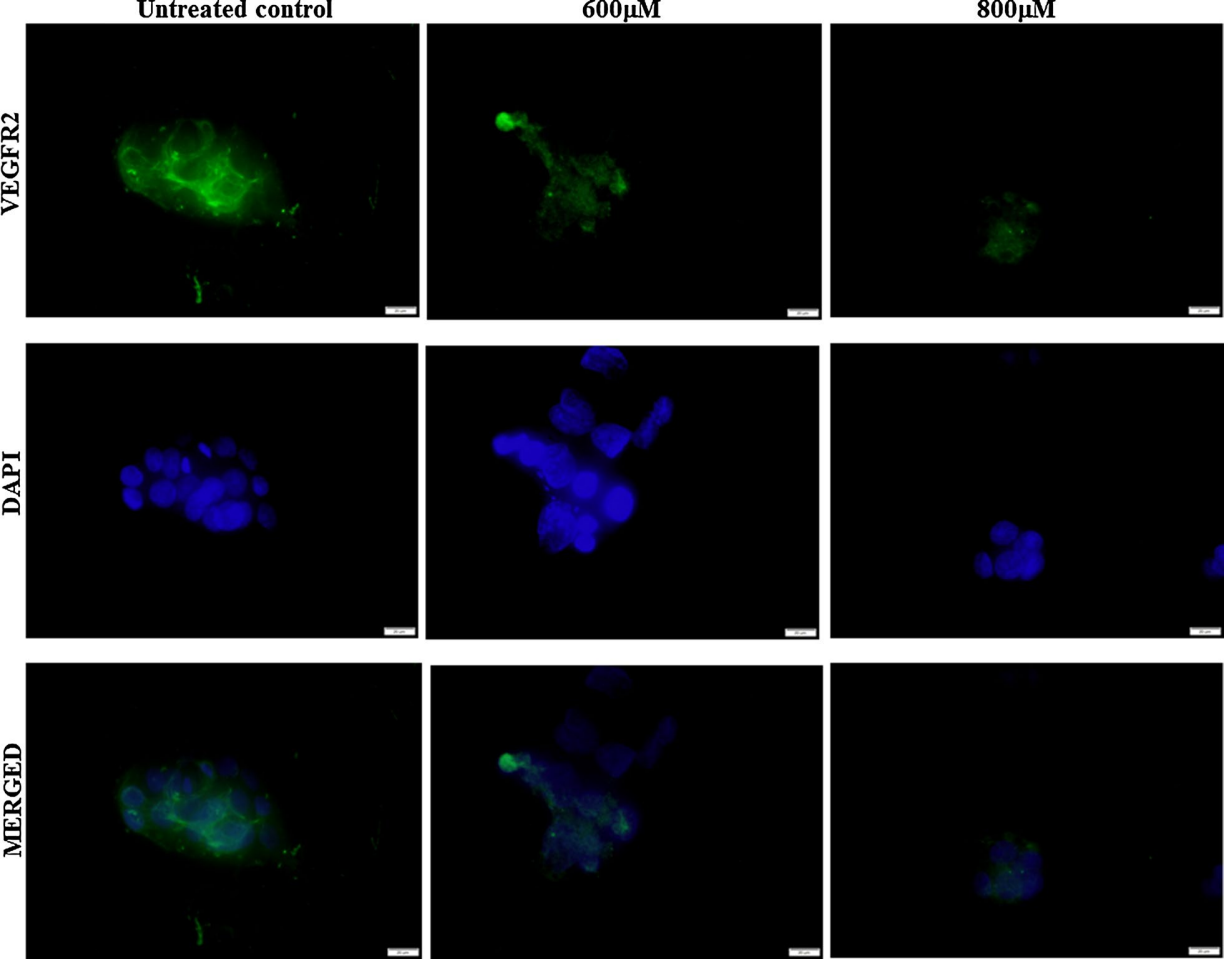
Representative images of immunofluorescence assay of pre-treated HEPG2 cells in two doses of *Matricaria recutita* L. extract (600 µM and 800 µM). Cells were immunostained with antibody against VEGFR2. VEGFR-positive cells were stained green (Alexa Fluor^®^ 488) and the nucleus stained blue (DAPI). Scale bar, 20 µm

Angiogenesis is central to many physiological conditions [29]. Vascular endothelial growth factor (VEGF) is a signal protein that stimulates the process of blood vessel formation through important cellular processes of vasculogenesis and angiogenesis through receptor tyrosine kinase VEGF receptors (VEGFRs). Multiple VEGFs (VEGF-A, VEGF-B, VEGF-C and VEGF-D) interact with VEGF receptors such as VEGFR1, VEGFR2 and VEGFR3 [50]. Here, we demonstrated that an increasing dose of the present extract inhibited the protein expression of VEGF (Fig. 2) and that was consistent with an immunofluorescence assay that showed the dose dependent decrease of VEGFR2 expression (Fig. 3).

The matrix metalloproteinases are zinc-dependent endopeptidases [51] of which MMP-9 is believed to be promoting angiogenesis [52]. The present extract dynamically down regulated the expression of MMP-9 substantiating the potential of the extract in regulating tumor angiogenesis. Protein expression analysis was performed using western blot to determine whether the present extract regulated the activity of MAPK/AKT cascades including AKT, ERK1/2 MAPK and p38 MAPK. As shown in Fig. 2, the extract substantially induced the activation of AKT and ERK1/2 MAPK and had no significant effect on phosphorylation of p38 MAPK. The tested chamomile extract inhibited the activation of AKT/ERK pathway measured by the western blot analysis of p-AKT and p-ERK1/2 (Fig. 2). Interestingly, the levels of total AKT was also down regulated at higher doses. Studies have reported that deprivation of VEGF or blockade of the VEGF signal transduction cascade with the VEGF tyrosine kinase inhibitor K787/ZK222584 resulted in a specific decrease of AKT protein level; subsequent cellular stimulation with VEGF rescued AKT stability in endothelial cells [53]. Chamomile extract used here could be a direct target for VEGF that caused the decrease of AKT protein expression and it significantly inhibited the level of VEGFR2 in HEPG2 cells representing the blockade of the VEGF signaling pathway. Similarily, *Rhazya strict* (Harmal) has recently been shown to possess potent antiangiogenic and antiproliferative activities in vitro [54].

#### Inhibitory effect of *M. recutita *${\text{L.}}$ extract on sprouting of microvessels in rat aortic explants

Cell migration and invasion are crucial in physiological processes such as angiogenesis. Similar trend was observed in aortic ring assay (Additional file 2) where treatment with chamomile reduced sprouting microvessels in a dose-dependent fashion.

### Conclusions

Chamomile represents a potential in anti-cancer treatments.

## Limitations

A possible limitation could be the lack of in vivo studies that was not the focus of this work but could be done in a future study.

## Additional files

**Additional file 1.** DPPH radical scavenging activities of the tested *Matricaria recutita* L. extract. Description of data: The percentage inhibitions of DPPH scavenging activity of the extract.

**Additional file 2.** *Matricaria recutita* L. extract inhibits microvessels sprouting in aortic ring assay in a dose-dependent manner. **(a)** Representative micrographs of sprouting microvessels from aortic ring grown in the absence or presence of *M. recutita* L. extract with or without VEGF treatment. **(b)** Quantification of the number of the sprouting microvessels from aortic rings grown in the presence or absence of *M. recutita *L. extract with or without VEGF treatment. Description of data: *Matricaria recutita* L. extract inhibits microvessels sprouting in aortic ring assay in a dose-dependent manner.

## Abbreviation

SEM: standard error of the mean
