# Supplementary material for: Antioxidant and anticancer activities of chamomile (Matricaria recutita L.)
Source: BMC Res Notes. 2019 Jan 3;12:3. doi: 10.1186/s13104-018-3960-y (PMC6317209; doi:10.1186/s13104-018-3960-y)
Supplement: Supplementary file 1 — Additional file 1. DPPH radical scavenging activities of the tested Matricaria recutita L. extract. Description of data: The percentage inhibitions of DPPH scavenging activity of the extract. [file 13104_2018_3960_MOESM1_ESM.docx]

**File name: Additional file 1**

**Title of data: DPPH radical scavenging activities of the tested *Matricaria recutita* L. extract.**

**Description of data:** The percentage inhibitions of DPPH scavenging activity of the extract.


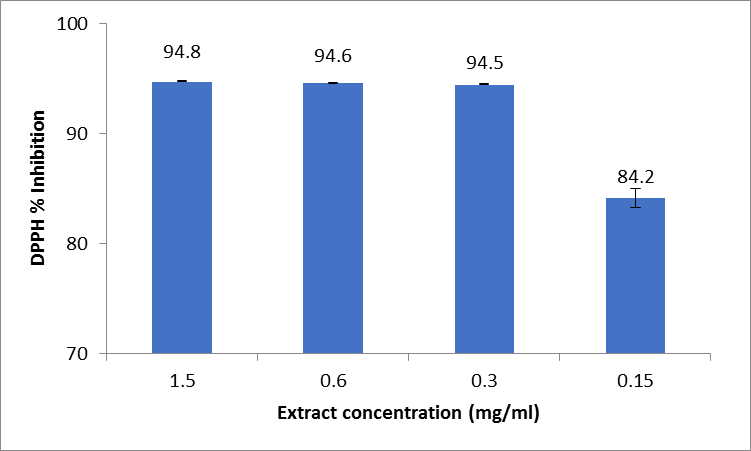


**Additional file 1.** DPPH radical scavenging activities of the tested *Matricaria recutita* L*.* extract.
