# Supplementary material for: Antioxidant and anticancer activities of chamomile (Matricaria recutita L.)
Source: BMC Res Notes. 2019 Jan 3;12:3. doi: 10.1186/s13104-018-3960-y (PMC6317209; doi:10.1186/s13104-018-3960-y)
Supplement: Supplementary file 2 — Additional file 2. Matricaria recutita L. extract inhibits microvessels sprouting in aortic ring assay in a dose-dependent manner. (a) Representative micrographs of sprouting microvessels from aortic ring grown in the absence or presence of M. recutita L. extract with or without VEGF treatment. (b) Quantification of the number of the sprouting microvessels from aortic rings grown in the presence or absence of M. recutita L. extract with or without VEGF treatment. Description of data: Matricaria recutita L. extract inhibits microvessels sprouting in aortic ring assay in a dose-dependent manner. [file 13104_2018_3960_MOESM2_ESM.docx]

**File name: Additional file2**

**Title of data:** *Matricaria recutita* L*.* extract inhibits microvessels sprouting in aortic ring assay in a dose-dependent manner. **(a)** Representative micrographs of sprouting microvessels from aortic ring grown in the absence or presence of *M. recutita* L. extract with or without VEGF treatment. **(b)** Quantification of the number of the sprouting microvessels from aortic rings grown in the presence or absence of *M. recutita* L. extract with or without VEGF treatment

**Description of data:** *Matricaria recutita* L*.* extract inhibits microvessels sprouting in aortic ring assay in a dose-dependent manner.

**Additional file 2.** *Matricaria recutita* L. extract inhibits microvessels sprouting in aortic ring assay in a dose-dependent manner. **(a)** Representative micrographs of sprouting microvessels from aortic ring grown in the absence or presence of *M. recutita* L*.* extract with or without VEGF treatment. **(b)** Quantification of the number of the sprouting microvessels from aortic rings grown in the presence or absence of *M. recutita* extract L. with or without VEGF treatment.
